# Professional Fraternity

**Published:** 1879-05-20

**Authors:** 


### EDITORIAL AND MISCELLANEOUS.

### *PROFESSIONAL FRATERNITY.*

The Session of the American Medical Association, recently held in this city, will long be remembered by those of the Profession who participated in its reunion. It was not only a great pleasure, but a high privilege to meet the members of this Body as professional brethren, and as noble, generous-hearted, courteous gentlemen. The unity of feeling, harmony of action, the earnestness and dignity of discussion and the entire *entente cordiale* that characterized the proceedings of the Sessions were most delightful and grateful to all present, and evinced that in true Medical Science, conflicting theories are rather for the purpose of eliciting and establishing a truth than for the bluster of a vain and dogmatic speculation. Science is broad, cosmopolitan and untramelled by prejudice in the vindication of its tenets. It strives to prove its positions, to demonstrate philosophical principles by its own liberal and true light, establishing them upon the solid basis of facts that cannot be controverted ; thus making the elucidation clear as noonday and satisfactory to those interested.

And it is the province of Medical Science to present an opposing front to all error within its ranks, to investigate every proposition with a jealous though impartial eye, and, conscious of the dignity and responsibility of its position, bring forward all its potent energy to refute and demolish blatant impositions upon suffering humanity.

We are glad to feel that our brethren seemed to be impressed more than ever with the truth that in every organization unanimity of purpose and action is a most forceful factor in its efficiency, and that zeal and harmony in the prosecution of its objects will follow such union, and cherish the kindliest fraternal feelings among the members of the body. Strife and dissension among individuals or bodies of men must always end in disastrous results to the success of a good cause and to the tender ties of friendship and brotherhood.

Amid the turmoils and harassing cares of life, these reunions of men of one heart, one object and one noble ambition, are most refreshing in a friendly aspect. We, as professional and editorial brethren, will feel all the better for having met each other face to face, given the hand a cordial grasp, and interchanged views upon the topics most interesting and important to us as *confreres* in Medical Journalism; thus cementing our action in the same grand and noble objects. Let us cordially exchange our respective journals, read them and profit by their contents, and encourage all the Profession to come up mightily to the help of the ever onward and upward movement in Medical Science.

As a social success, the convening of the American Medical Association in Atlanta was notable and most pleasant. The noble, hightoned gentlemen composing the Body were received with open hearts and hands by our best society, and left nothing but pleasing impressions upon all who met them ; and compliments from the press of Atlanta, and from all classes of citizens were numerous, and, we have no doubt, were sincerely uttered. Not only the Profession of the city, but our social circles also, enjoyed the presence of the brotherhood amongst us, and a host of kind wishes followed them to their respective homes.

Despite the many *bons mots* thrown at the Medical Profession, the remarks made in regard to its " peopling cemeteries," "humbugging the credulous," " making capital of human suffering," etc., it cannot be denied, and we say it not boastingly, but gratefully, that the " Doctors " are on a whole, favorites in social and family circles, and are noted for their kindness, courtesy and friendly confidence. God grant we may all endeavor to deserve in the best and highest sense the appreciative favor, the confidence and patronage of our friends !

The convening of the American Medical Association in Atlanta, May 6th, 1879, will be marked with a white stone. Farewell, my brethren ! may the Omnipotent Sovereign of all nations of the earth spare our lives, and may we meet again in the great metropolis of the American Union, in 1880. P.

